# Dengue Virus Infection in Africa

**DOI:** 10.3201/eid1708.101515

**Published:** 2011-08

**Authors:** Ananda Amarasinghe, Joel N. Kuritsky, G. William Letson, Harold S. Margolis

**Affiliations:** Author affiliations: International Vaccine Institute, Seoul, South Korea (A. Amarasinghe, J.N. Kuritsky, G.W. Letson);; Centers for Disease Control and Prevention, San Juan, Puerto Rico, USA (H.S. Margolis)

**Keywords:** Africa, arbovirus infections, dengue, viruses, infectious disease transmission, incidence, travel medicine, synopsis

## Abstract

Reported incidence of dengue has increased worldwide in recent decades, but little is known about its incidence in Africa. During 1960–2010, a total of 22 countries in Africa reported sporadic cases or outbreaks of dengue; 12 other countries in Africa reported dengue only in travelers. The presence of disease and high prevalence of antibody to dengue virus in limited serologic surveys suggest endemic dengue virus infection in all or many parts of Africa. Dengue is likely underrecognized and underreported in Africa because of low awareness by health care providers, other prevalent febrile illnesses, and lack of diagnostic testing and systematic surveillance. Other hypotheses to explain low reported numbers of cases include cross-protection from other endemic flavivirus infections, genetic host factors protecting against infection or disease, and low vector competence and transmission efficiency. Population-based studies of febrile illness are needed to determine the epidemiology and true incidence of dengue in Africa.

Dengue has emerged in recent decades as a worldwide public health problem, particularly in the Asia–Pacific and Americas–Caribbean regions (*1**–**3*). In Africa, the epidemiology and public health effect of dengue is not clear. *Aedes* spp. mosquitoes are widely distributed in Africa and can serve as vectors of dengue virus (DENV). When their distribution is combined with rapid population growth, unplanned urbanization, and increased international travel, extensive transmission of DENV is likely in Africa (Figure) (*2**,**4*). Over the past 5 decades, cases of epidemic or sporadic dengue have been reported in many countries in sub-Saharan Africa (*5*). Although sporadic cases of dengue hemorrhagic fever (DHF) have been reported in a few countries in Africa, no outbreaks have been reported (*1*). However, when compared with the Asia–Pacific and Americas–Caribbean regions, the epidemiology and effect of dengue in Africa has not been defined. A dengue outbreak in Cape Verde was recently reported (>3,000 cases), and the reappearance of dengue in Senegal after 20 years was also reported (*6*). To estimate the extent of DENV infection and dengue in Africa, we reviewed published literature and other sources for reports of this disease in persons 1iving in or traveling to this region.

**Figure:**
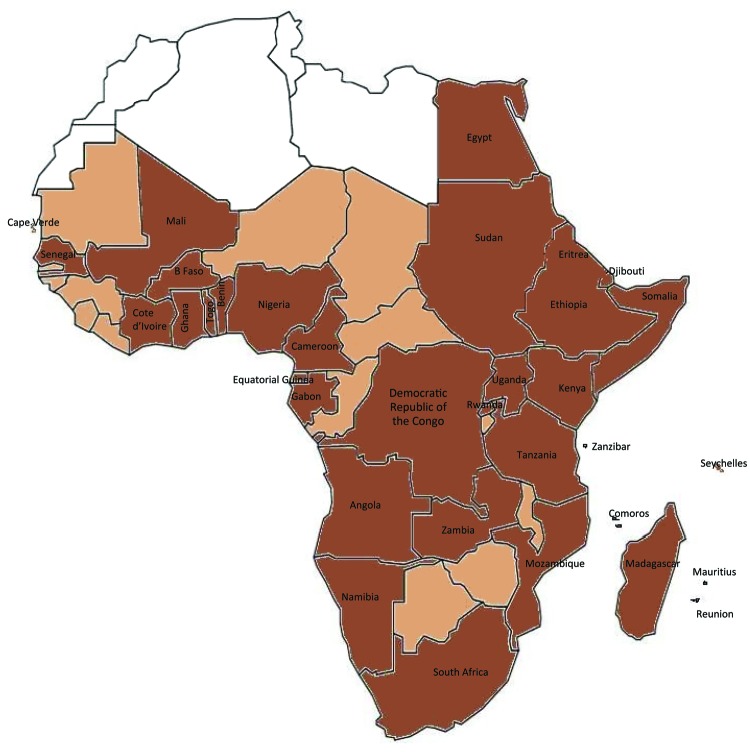
Dengue and *Aedes aegypti* mosquitoes in Africa. Brown indicates 34 countries in which dengue has been reported, including dengue reported only in travelers, and *Ae*. *aegypti* mosquitoes. Light brown indicates 13 countries (Mauritania, The Gambia, Guinea-Bissau, Guinea, Sierra Leone, Liberia, Niger, Chad, Central African Republic, Republic of the Congo, Malawi, Zimbabwe, and Botswana) in which dengue has not been reported but that have *Ae. aegypti* mosquitoes. White indicates 5 countries (Western Sahara, Morocco, Algeria, Tunisia, and Libya) for which data for dengue and *Ae. aegypti* mosquitoes are not available.

## Search Methods

Published, peer-reviewed literature, published and unpublished country reports, and the World Health Organization (WHO) library database, including Dengue Net, were reviewed for 1960–2010 for the key terms “dengue” and “Africa.” In addition, we examined peer-reviewed published literature and other sources to determine the extent of disease among travelers returning from Africa. We searched for publications in English by using MEDLINE and EMBASE electronic databases, Euro Surveillance, and ProMED-mail posts. A review for dengue reports in languages other than English did not find any reports that would change the conclusions of this article.

Additionally, references in each paper identified during searches were checked. Those references not already identified by the search were reviewed. Abstracts presented at international forums were included if they contained epidemiologic, entomologic, or virologic data pertaining to dengue in Africa.

## Reported Dengue in Africa

Dengue was reported in Africa in the late 19th and early 20th centuries. Epidemics were reported in Zanzibar (1823, 1870), Burkina Faso (1925), Egypt (1887, 1927), South Africa (1926–1927), and Senegal (1927–1928) (*1**,**5**,**7*). The epidemic in South Africa was confirmed by retrospective neutralizing antibody testing in the mid-1950s, but the other reported epidemics were not laboratory confirmed and therefore may not have been dengue. During the 50 years from 1960 through 2010, twenty laboratory-confirmed dengue outbreaks were reported in 15 countries in Africa; most were from eastern Africa. Nearly 300,000 cases were reported in 5 large epidemics in the Seychelles (1977–1979), Réunion Island (1977–1978), Djibouti (1992–1993), Comoros (1992–1993), and Cape Verde (2009) (*6**–**9*). DENV was first isolated in Africa in Nigeria in the 1960s (*10*). Subsequently, all 4 DENV serotypes have been isolated in Africa (*1*). DENV-2 has been reported to cause most epidemics, followed by DENV-1 (*8**,**9*).

Available data strongly suggest that DENV transmission is endemic to 34 countries in all regions of Africa (Figure, Table). Of these countries, 22 have reported local disease transmission, 20 have reported laboratory-confirmed cases, and 2 (Egypt and Zanzibar) have reported only clinical cases that were not laboratory confirmed. In the remaining 12 countries, dengue was diagnosed only for travelers returning to countries to which dengue was not endemic but never reported as occurring locally in these 12 countries (Table).

**Table:** Countries in Africa with evidence of dengue virus transmission*

Type and country	Year	Serotype
Locally acquired, n = 7		
Cape Verde	2009†	3
Egypt	1779, 1887, 1927	Unknown
Eritrea	2005	Unknown
Mauritius	2009	Unknown
Réunion	1977–1978†	2
Seychelles	1977–1979†	2
Sudan	1984–1986	1 and 2
Locally and travel acquired, n = 15		
Angola	1986, 1999–2002‡	Unknown
Burkina Faso	1925	Unknown
	1983–1986	2
	2003–2004,§ 2007‡	Unknown
Cameroon	1987–1993,§ 1999–2002,‡ 2000–2003,§ 2006‡	Unknown
Comoros	1943–1948	Unknown
	1984, 1992–1993†	1 and 2
Djibouti	1991–1992†	2
Côte d’Ivoire	1982	2
	1998	1
	1999–2002‡	Unknown
	2008	3
Ghana	1932, 1987–1993§	Unknown
	2002–2005	2
Kenya	1982	2
	1984–1986	1 and 2
Madagascar	1943–1948	Unknown
	2006	1
Mozambique	1984–1985†	3
Nigeria	1964–1968	1
Senegal	1928	Unknown
	1979	1
	1980–1985	2 and 4
	1990, 1999	2
	2007‡	Unknown
	2009	3
Somalia	1982, 1985–1987	2
	1992–1993	2 and 3
South Africa	1927†	1
Zanzibar	1823, 1870, 2010‡	Unknown
Travel/expatriate acquired, n = 12		
Benin	1987–1993§	Unknown
DRC	1999–2001,‡ 2007§	Unknown
Ethiopia	1999–2002,‡ 2007§	Unknown
Equatorial Guinea	1999–2002‡	Unknown
Gabon	1999–2002‡	Unknown
Mali	2008	2
Namibia	1999–2002,‡ 2006‡	Unknown
Rwanda	1987–1993§	Unknown
Tanzania	1987–1993,§ 1999–2002,‡ 2006,‡ 2010‡	Unknown
Togo	1987–1993,§ 1999–2002‡	Unknown
Uganda	1999–2002‡	Unknown
Zambia	1987–1993§	Unknown

*DRC, Democratic Republic of the Congo. †Large local outbreaks. ‡TropNet Europ Network (www.tropnet.net) and ProMED mail (www.promedmail.org). §Seroprevalence study.

## Dengue among Travelers/Expatriates Returning from Africa

The European Network on Imported Infectious Disease Surveillance and other published data have reported 27 countries in Africa as locations where travelers/expatriates from regions to which dengue was not endemic acquired dengue (Table) (*11**–**15*). Among travel-acquired dengue cases reported among persons from Europe, only 2%–8% had visited Africa (*11**–**14*). Although 54%–61% and 25%–31% of returning travelers with dengue returned from Asia and Latin America, respectively, Africa seems somewhat underrepresented with respect to dengue. However, this finding is not the result of a paucity of visits among travelers from countries to which dengue is not endemic.

Wilson et al. reported for the GeoSentinel Surveillance Network of the International Society of Travel Medicine and the Centers for Disease Control and Prevention that travelers reporting illness have disproportionately visited Africa (*15*). Febrile illness was more frequently reported for travelers to sub-Saharan Africa (371 febrile illnesses/1,000 patients) than to any other region, followed by Southeast Asia (248/1,000) and South America (143/1,000) (*11*). Similar to reported global dengue endemicity patterns by region, travelers with dengue came more frequently from Southeast Asia and South America than Africa (*11*).

## Prevalence of DENV Infection in Africa

Although outbreaks of dengue have been reported, data on incidence or prevalence are not available for Africa. A study in Nigeria determined the prevalence of flavivirus infections among 1,816 children and adults from urban and rural areas in samples obtained mainly during the early 1970s. Virus-specific hemagglutination inhibition testing showed that the prevalence of immunity was 38% for DENV-1 infection, 45% for DENV-2 infection, 43% for yellow fever virus infection, and 49% for West Nile virus infection (*16*). Serum specimens were also tested by suckling mouse neutralization of DENV-2. The authors concluded that because a high proportion of specimens with antibody to DENV-2 were confirmed by neutralization and because many had only monotypic antibody, the prevalence results were not likely confounded by cross-reactive antibody to other flaviviruses. In addition, this study showed an increase in prevalence of antibodies against DENV with age, which suggests endemic infection (*16*). Collenberg et al. reported that the prevalence of antibodies against DENV determined by immunoglobulin G indirect ELISA among a sample of pregnant women and blood donors (n = 683) was 26.3% in rural settings and 36.5% in urban settings in Burkina Faso (*17*). However, in Cameroon, the prevalence of antibodies against DENV determined by neutralization testing among 256 adults was only 12.5% (*18*).

The prevalence of DENV infection found by these studies was considerably lower than that found in dengue-endemic areas of Asia and the Americas (*19*). However, it is difficult to generalize from the small number of studies in Africa because they had small sample sizes and noncomparative age groups. Although the testing method used for the studies in Nigeria and Cameroon studies would minimize overestimation of DENV infection prevalence because of cross-reactive antibodies to other flavivirus infections or yellow fever vaccination, use of an immunoglobulin G ELISA in the Burkina Faso study did not differentiate these infections.

## Underrecognition of Dengue in Africa

In regions to which malaria is endemic, >70% of febrile illnesses are treated as presumptive malaria, often without proper medical examination and a laboratory diagnosis (*20**,**21*). In a setting where diagnostic testing is conducted, such as the GeoSentinel Surveillance Network, malaria was found to be the predominant cause of systemic febrile illness among travelers returning from sub-Saharan Africa (622/1,000 patients) compared with dengue (7/1,000) (*11*). This finding is not unexpected because malaria is more endemic to Africa than other febrile illnesses. However, overdiagnosis of malaria in areas of low transmission is well documented, and overestimation by clinical diagnosis is ≈61% (*20**,**21*). Many patients in Africa with fever are designated as having fever of unknown origin or malaria and remain without a diagnosis even if they fail to respond to antimalarial drugs. Under these prevailing practices, there is a real potential of misdiagnosing dengue as malaria.

In disease-endemic countries in the Asia–Pacific and Americas–Caribbean regions, dengue accounts for 3%–11% of febrile illnesses (*19*). Although dengue is well recognized as a public health problem in these regions, underreporting is common. Capture–recapture studies in Puerto Rico showed that the degree of underreporting and reporting in a passive surveillance system was ≈60% (*22*). Wichmann et al. showed that in Thailand and Cambodia underreporting of dengue was 1.4–9.6-fold (*23*). A virologic study conducted in the Sudan among 100 consecutive hospitalized patients with fever reported that 21 cases were caused by DENV infection (*24*). Coupled with the bias toward classifying most febrile illness as malaria, we expect that there is substantial underrecognition and underreporting of dengue in Africa.

During the 18th and 19th centuries, dengue was recognized almost exclusively among colonial settlers and military forces in Asia and the Americas and not among the local population, probably as a consequence of inadequate clinical investigation and surveillance (*25*). Similarly, except for some reported local outbreaks, many cases of dengue in Africa are more frequently reported among travelers than among the local population, which suggests lack of awareness, diagnostic facilities, and surveillance. In addition, travelers with febrile illness are frequently given a misdiagnosis of malaria; a rate of misdiagnosis as high as 77% has been reported (*20*). Of 27 countries in Africa where travelers/expatriates acquired dengue, only 15 have reported local disease transmission (Table). Therefore, travel-acquired dengue appears to serve as a proxy for identifying the underrecognition of dengue in Africa.

## Factors Potentially Affecting Sustained Transmission of DENV in Africa

### Vector Efficiency

*Aedes aegypti* mosquitoes, the principal DENV vector, originated in Africa and spread to other countries in Africa and other tropical countries in the 17th and 18th centuries (*1**,**3*). Several other *Aedes* species mosquitoes, including *Ae. albopictus*, *Ae. africanus*, and *Ae. luteocephalus*, are found in Africa and are potential DENV vectors.

Urbanization is a major factor in facilitating the increase of *Aedes* spp. mosquito populations (*1*). Accumulation of nonbiodegradable, human-made containers in and around living areas has provided the aquatic environment required by these mosquitoes (*25*). Since the 1950s, a 3-fold increase in urban human population density has occurred in Africa; larger increases have occurred in Asia and the Americas (*4*). With these demographic changes and subsequent increases in *Aedes* spp. populations, increased DENV transmission is likely to occur in Africa. For example, in Ghana, *Aedes* spp. mosquito densities and biting rates seem sufficient to result in outbreaks of yellow fever and dengue (*26*).

Susceptibility of different strains of *Aedes* spp. mosquitoes to DENV has been shown to vary geographically, and this variability may have implications for DENV transmission and the epidemiology of the disease in Africa. Mosquito strains in Africa have shown uniformly low susceptibility to all 4 DENV serotypes in laboratory settings (*27**–**29*). In addition, it has been well documented that there are different susceptibilities of the vector to different DENV genotypes; *Ae. aegypti* mosquitoes tend to be more susceptible to infection with DENV-2 of the Southeast Asian genotype than to the American genotype (*30*). Similar findings have been described for yellow fever virus, and the reduced vector competence of strains of *Ae. aegypti* mosquitoes from Asia has been suggested as an explanation for the absence of this disease in Asia (*2**,**31*). Reduced vector competence for DENV infection in Africa may be an explanation for some of the apparent low prevalence of DENV infection in Africa, although this explanation must be confirmed in appropriate studies.

*Ae. albopictus* mosquitoes are also potential DENV vectors in Africa where they are considered more anthropophilic than *Ae. aegypti* mosquitoes, more susceptible to DENV infection, and are responsible for some dengue outbreaks in Africa (*29**,**32**–**33*). However, similar to studies with *Ae. aegypti* mosquitoes, experimental studies with *Ae. albopictus* mosquitoes have demonstrated that geographic variations in susceptibility to DENV infection occur among different species (*28**,**29*). Furthermore, *Ae. albopictus* mosquitoes are believed to be less efficient as an epidemic vector largely because of their differences in host preferences and reduced vector competence, which decreases the probability of sustained disease transmission (*34*). Thus, appropriate ecologic studies are needed in Africa to determine the relative roles of each species in transmission of DENV.

### Virus Infectivity

Dengue is caused by 4 genetically related but antigenically different viruses, and although it is uncertain where DENV evolved, maintenance of all 4 serotypes in enzootic cycles in Africa suggests that a progenitor virus most likely originated in Africa (*1*). Despite the apparent origin of DENV in Africa hundreds of years ago, the more recent reported outbreaks appear to be the result of virus introductions from Southeast Asia or the western Pacific region and not the result of spillover from forest transmission cycles (*25*).

Vasilakis et al. reported that the rate of evolutionary change and pattern of natural selection are similar among endemic and sylvatic DENVs and suggested possible future reemergence of DENV from the sylvatic cycle (*35*). Recent experimental evidence suggests that emergence of endemic DENV-2 from sylvatic progenitors may not have required adaptation to replicate efficiently in humans, implying that sylvatic DENV-2 may reemerge (*35*). Existence of a silent zoonotic transmission cycle affords a potential mechanism for emergence of dengue in human populations and for selection of virus variants with altered host range and vector relationships (*25*).

### Host Susceptibility

Host genetic factors influencing pathogenesis have been suggested to account for some variability in susceptibility of DENV infection and disease expression among different races. Halstead et al. provided evidence of a dengue resistance gene in the black population (*36*). During the 1981 and 1997 dengue epidemics in Cuba, blacks were hospitalized with DHF/dengue shock syndrome at lower rates than whites (*37*). This potential decreased susceptibility to severe disease among the black population and similar observations in Haiti have been used to support the hypothesis that specific genomic difference among different racial groups is a risk factor for DHF (*36**,**38*). This hypothesis may provide an explanation for the observation that, to our knowledge, outbreaks of DHF/dengue shock syndrome have not been reported in Africa.

Other prevailing diseases in Africa could provide another hypothesis to explain the apparently low incidence of dengue. Malaria, tuberculosis, and HIV infections are endemic to many parts of Africa. Prevailing socioeconomic and environmental factors may make populations in Africa more vulnerable to these diseases than to dengue. Monath (*31*) and Gubler (*2*) hypothesized that immunologic cross-protection from heterotypic antibodies to other flavivirus infections (DENV and Japanese encephalitis virus) could explain the absence of yellow fever virus in Asia. A similar argument could be made to explain the low rate of DENV infection caused by cross-protection from other endemic flaviviruses in Africa, but the extent to which it may exist is unknown.

## Conclusions

Dengue is a highly underrecognized and underreported disease even in areas of the world where there is a high level of public health and clinical awareness and diagnostic capacity. In Africa, most febrile illness is not assessed by laboratory diagnostics and is assumed to be malaria. Sustained, systematic surveillance for dengue-like illness combined with laboratory diagnostics and education of health care providers has been the source of the information about the public health role of dengue in Asia and the Americas. This surveillance is needed in Africa to determine the epidemiology and public health role of dengue.

The 2004 WHO Global Epidemiology of Infectious Diseases Study estimated that 2.4% of global DHF cases occurred in Africa and that 20% of the population in Africa was at risk for dengue (*39*). However, because these estimates were only for DHF and not dengue fever (DF), a conservative approach to estimate DF in Africa would be to apply the expected DHF to DF ratio of 1% to 5% to this WHO study estimate. Thus, 0.2–1.0 million cases of DF could be expected to occur in Africa on the basis of WHO estimates of 10,000 cases of DHF in Africa, although no DHF outbreaks have been reported.

Although there is some uncertainty about estimates of cases provided by various sources, these estimates provide a strong argument that DENV transmission is present in Africa but likely underreported. Reported outbreaks and dengue acquired by travelers to Africa from regions to which dengue is not endemic indicate that local transmission of DENV occurs in Africa. Furthermore, the apparent increase of dengue in the region is the result of an increase in the disease, consequence of improved disease reporting, or both. Nevertheless, the epidemiology of DENV transmission and the incidence of dengue in Africa are poorly defined.

Dengue is usually not among the differential diagnoses of acute febrile illness in Africa. Reasons for this lack of inclusion are as follows: 1) malaria is the most prominent endemic febrile illness in Africa and does not require complex clinical and laboratory diagnostic facilities; 2) a low awareness of dengue may contribute to health care workers not considering the disease; 3) dengue is not a reportable disease in most countries in Africa; 4) dengue surveillance and diagnostics are not widely and consistently available throughout Africa; and 5) funding for surveillance and other research activities pertaining to dengue in Africa are limited (*8**,**9*). For these reasons, improved surveillance and laboratory diagnosis of fevers in Africa is a priority and first step in assessing the incidence of dengue in Africa.

Whether populations in Africa are susceptible to DENV infection and disease at rates comparable with those in populations in Asia or the Americas and the true incidence of dengue in these countries cannot be determined from data obtained from occasional reports of disease outbreaks. Given that safe and effective dengue vaccines should become available within the next decade (*40*), questions regarding dengue incidence and epidemiology in Africa must be answered by using appropriately designed surveillance studies. Studies to determine the extent of DENV infection among persons of all ages with febrile illness could be included in other studies (e.g., malaria) being conducted in the region and would provide answers to speculation about dengue in Africa that has existed for many years.
